# Supplementary material for: Cluster tendency assessment in neuronal spike data
Source: PLoS One. 2019 Nov 12;14(11):e0224547. doi: 10.1371/journal.pone.0224547 (PMC6850537; doi:10.1371/journal.pone.0224547)
Supplement: S1 Appendix — (PDF) [file pone.0224547.s001.pdf]

	**Algorithm: VAT**
**In**	D, $n \times n$ matrix of dissimilarities: $D = D^T$; $d_{ij} \ge 0 \ \forall\, i, j$; $d_{ii} = 0 \ \forall i$
**Set**	$K = \{1, 2, \ldots, n\}$: $I = J = \varnothing$:<br>Select $(i, j) \in \arg\max\{D_{st} : s \in K, t \in K\}$<br>$P(1) = i$: $I = \{i\}$: $j = K - \{i\}$<br>% Initialize MST at either end of edge with largest weight in D
**For** m = 2,…, n **do**: select $(i, j) \in \arg\min\{D_{st} : s \in I, t \in J\}$<br>Select $(i, j) \in \arg\min\{D_{st} : s \in I, t \in J\}$<br>$P(m) = j$: $I = I \cup \{j\}$: $J = J - \{i\}$: $d_{m-1} = d_{ij}$	
**For** $1 \le i, j \le n$ **do**:<br>$[D^*]_{ij} = [D]_{P(i)P(j)}$	
**Out**	VAT reordered dissimilarities D*: arrays P, d<br>% Create VAT RDI I(D*) using D*
	**Algorithm: iVAT**
**In**	D* **=**VAT reordered dissimilarity matrix: D'* = [0]
**For** k = 2 to n **do**:<br>$j = \underset{r=1,\ldots k-1}{\arg\min} \{D^*_{kr}\}$<br>$D'^*_{kc} = D^*_{kc}$; $c = j$<br>$D'^*_{kc} = \max\{D^*_{kj}, D'^*_{jc}\}$; $c = 1, \ldots, k-1; c \ne j$	
**For** $j = 2, \ldots, n; i \ne j$:<br>$D'^*_{ji} = D'^*_{ij}$	
**Out**	**iVAT** Reordered dissimilarities D'*<br>% Create iVAT RDI I(D'*) using D'*
