# Supplementary figures and images for: Cluster tendency assessment in neuronal spike data
Source: PLoS One. 2019 Nov 12;14(11):e0224547. doi: 10.1371/journal.pone.0224547 (PMC6850537; doi:10.1371/journal.pone.0224547)

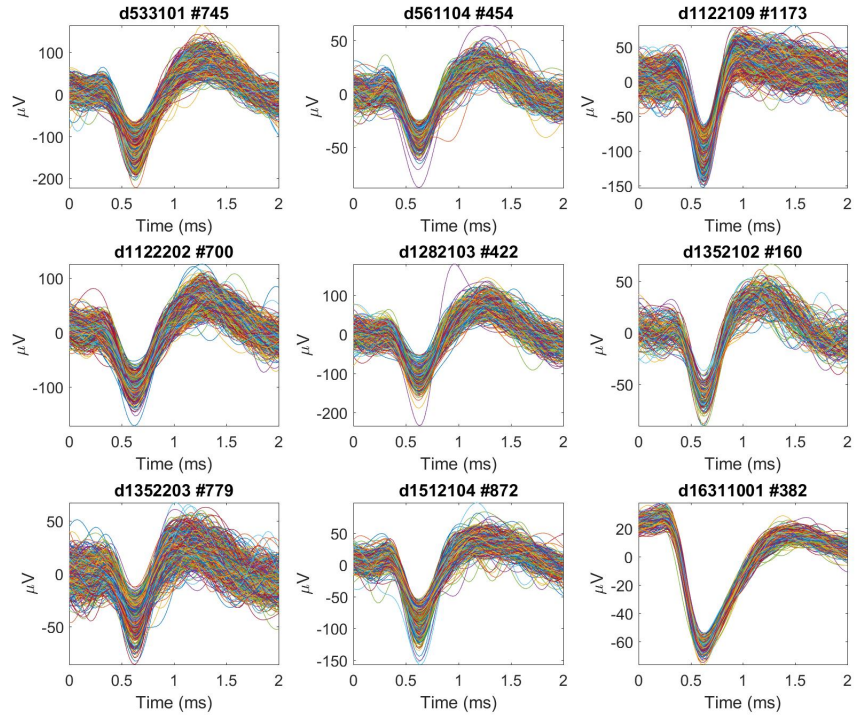

Supplement: S1 Fig — Each subplot title displays the label of the experiment in Henze2009 dataset and the number of spikes in each subset: e.g., #745 means there are 745 waveforms in the sample. (PDF) [file pone.0224547.s002.pdf]
