# Supplementary material for: Cluster tendency assessment in neuronal spike data
Source: PLoS One. 2019 Nov 12;14(11):e0224547. doi: 10.1371/journal.pone.0224547 (PMC6850537; doi:10.1371/journal.pone.0224547)
Supplement: S2 Fig — Each subset is generated from a template taken from real recordings as described in Material and Methods section. All the waveforms are in black and the average waveforms of subsets are in color. The color of the average waveforms doesn’t imply any order or significance. The colormap is Parula in MATLAB. The mixtures in subplots denoted by a yellow star label are the ones displayed as representative in the manuscript Figs 1 to 5. The one subplot with a red star is the mixture investigated in Fig 6. (PDF) [file pone.0224547.s003.pdf]

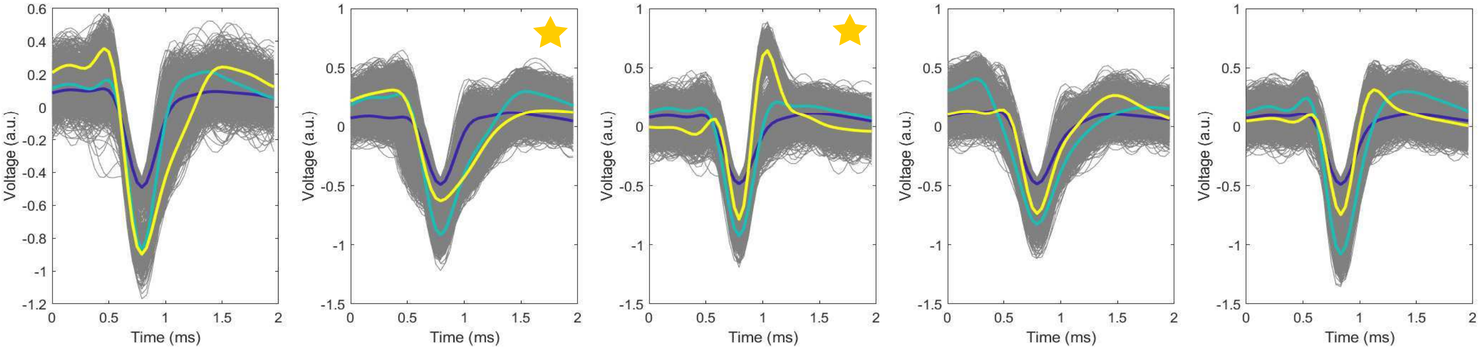

(a) Five mixtures of spikes, each including 3 subsets of spikes.

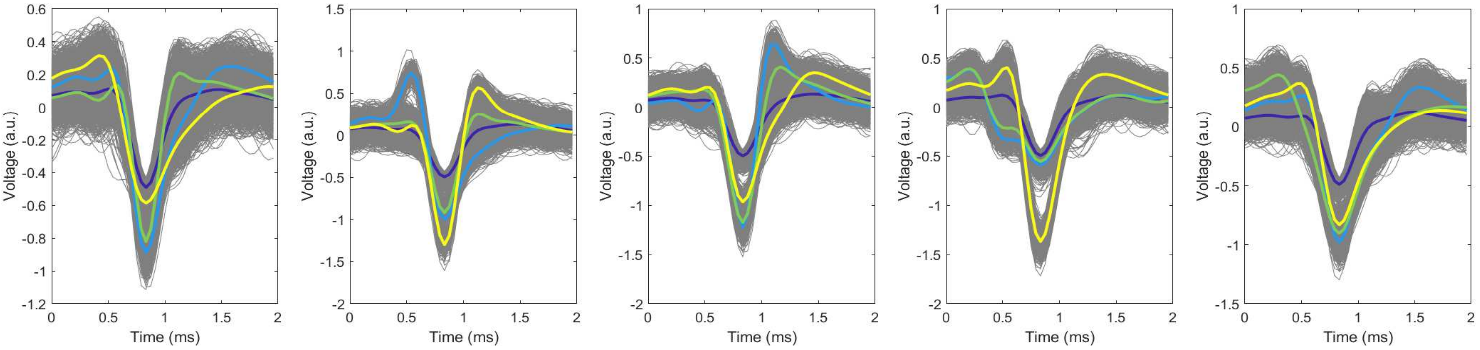

(b) Five mixtures of spikes, each including 4 subsets of spikes.

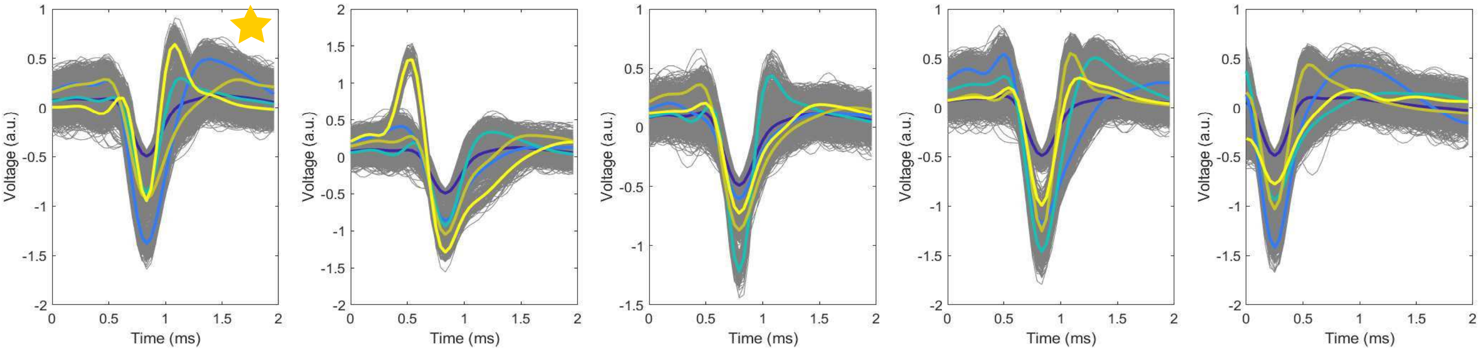

(c) Five mixtures of spikes, each including 5 subsets of spikes.

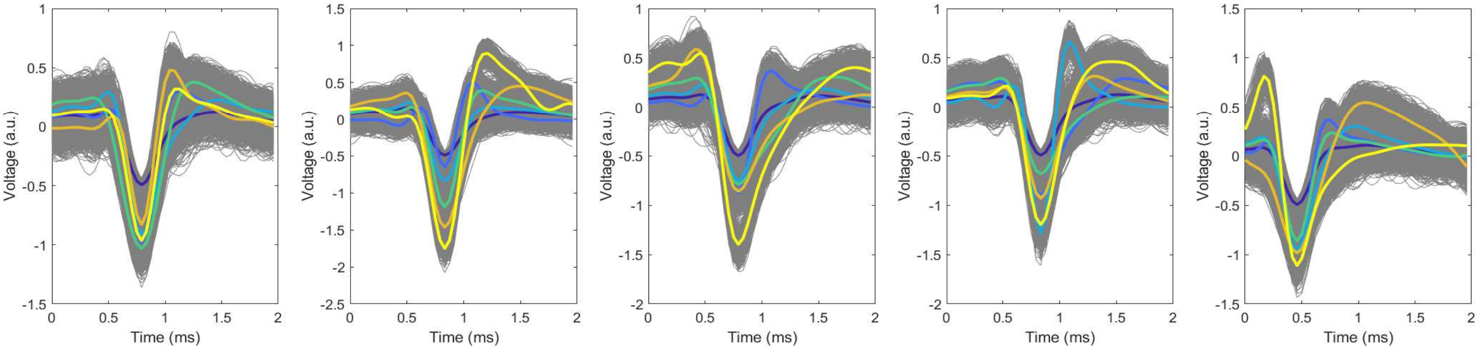

(d) Five mixtures of spikes, each including 6 subsets of spikes.

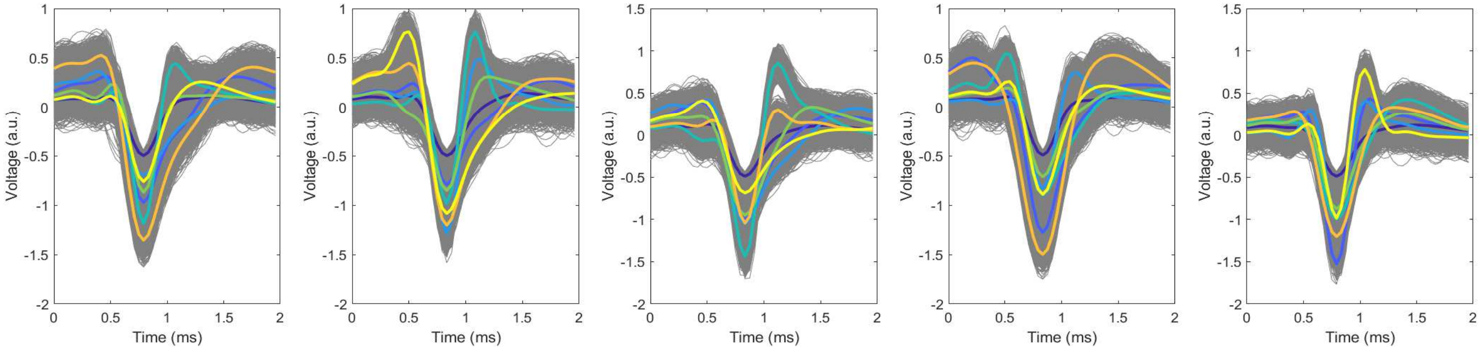


(e) Five mixtures of spikes, each including 7 subsets of spikes.

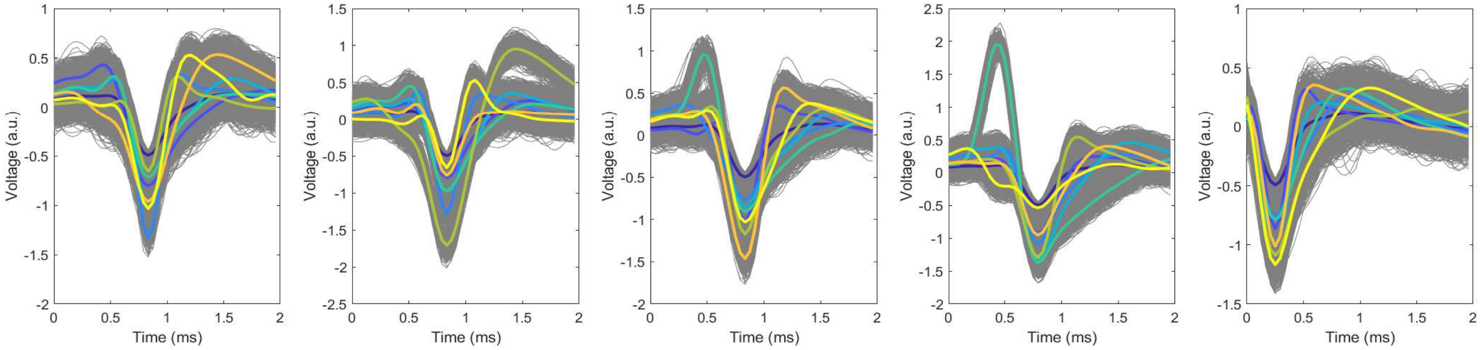


(f) Five mixtures of spikes, each including 8 subsets of spikes.

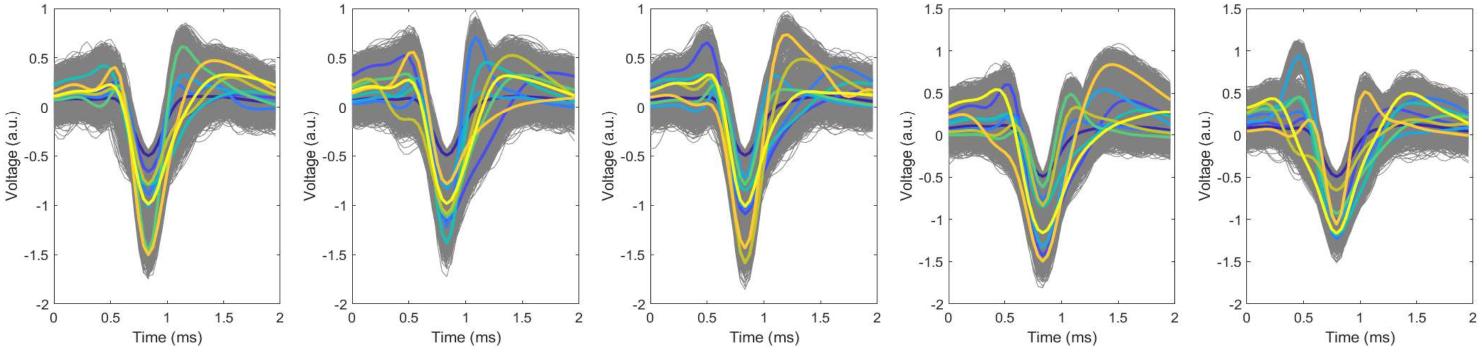


(g) Five mixtures of spikes, each including 9 subsets of spikes.

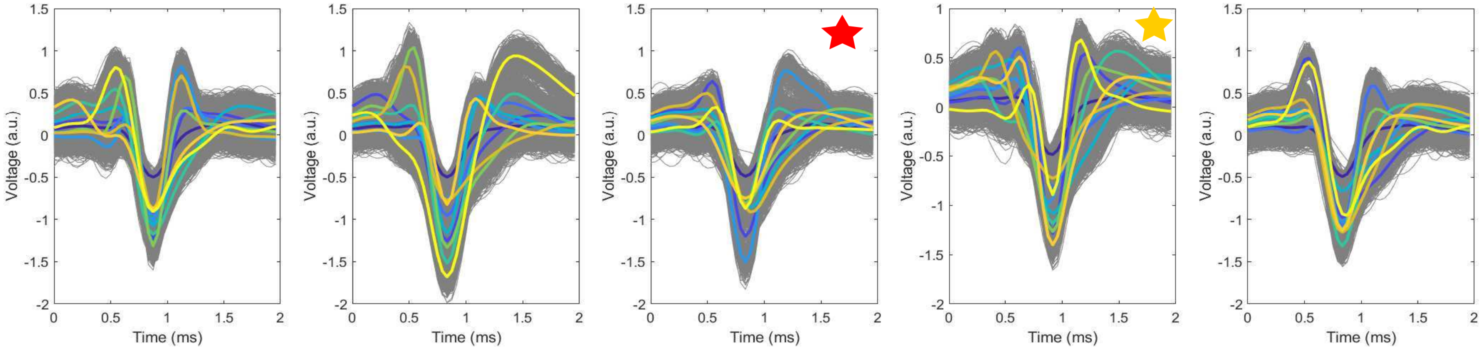


(h) Five mixtures of spikes, each including 10 subsets of spikes.

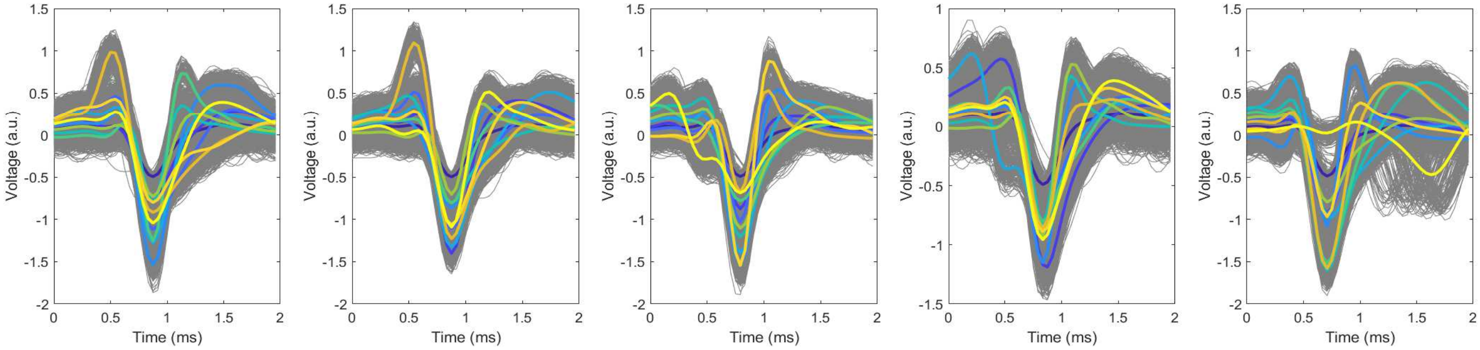


(i) Five mixtures of spikes, each including 11 subsets of spikes.

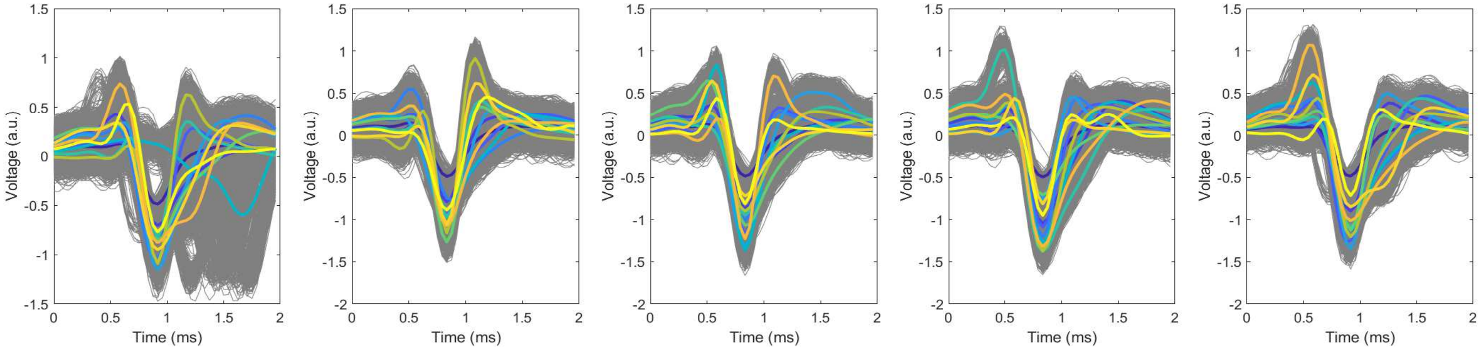


(j) Five mixtures of spikes, each including 12 subsets of spikes.

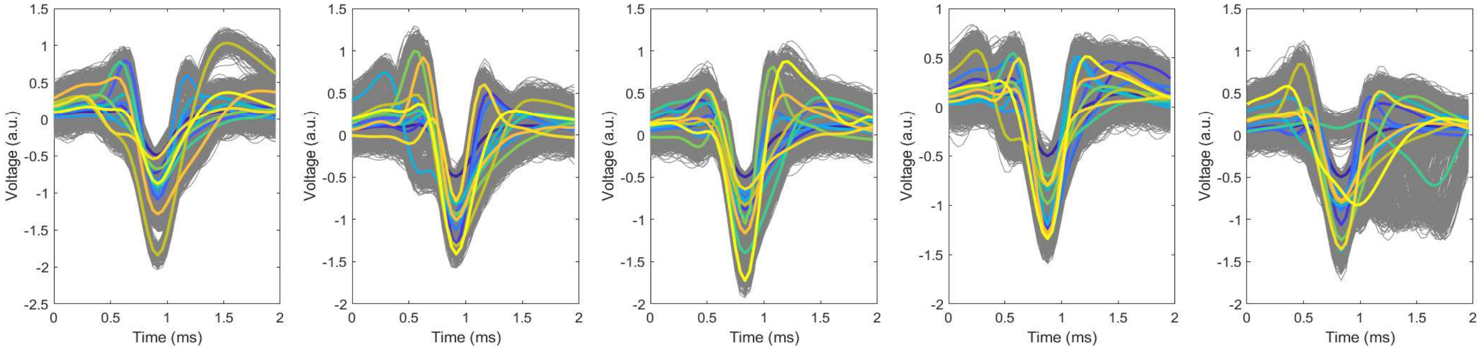


(k) Five mixtures of spikes, each including 13 subsets of spikes.

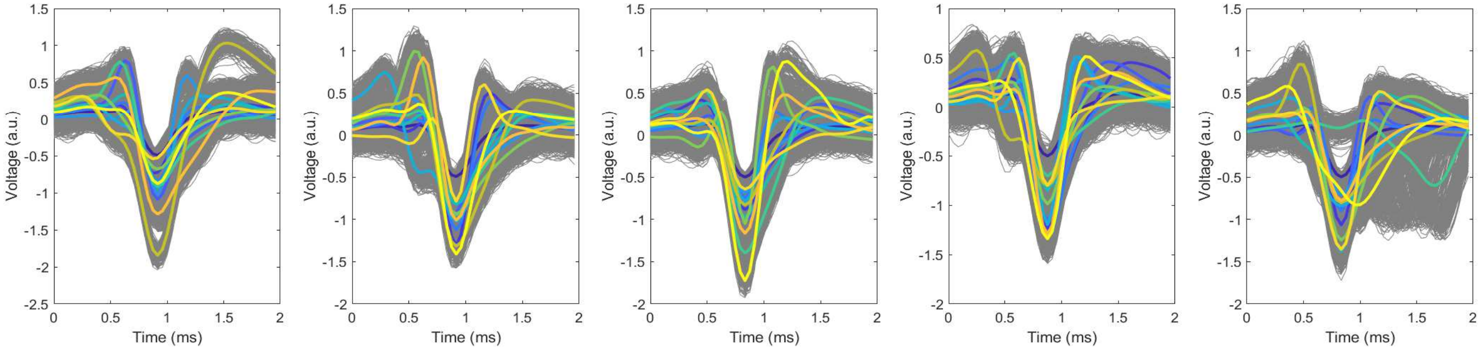


(l) Five mixtures of spikes, each including 14 subsets of spikes.

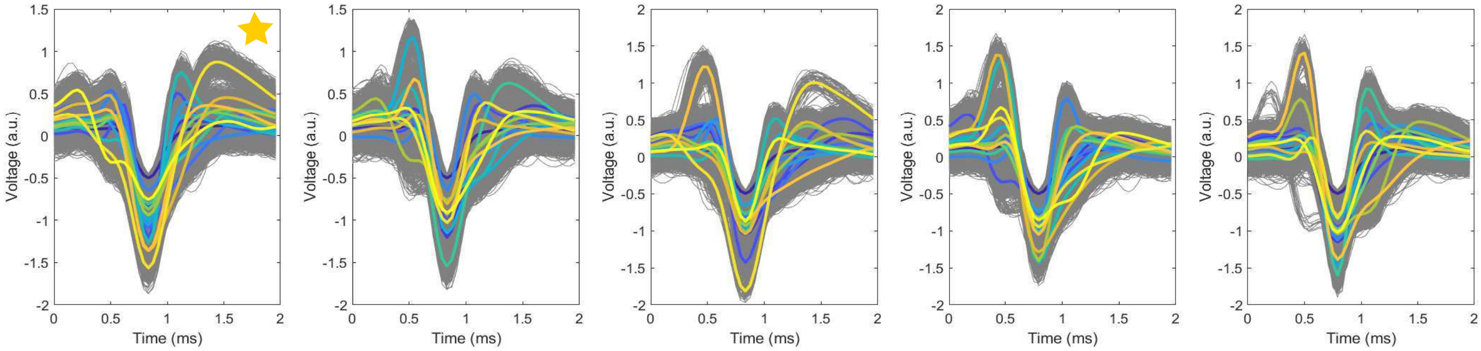


(m) Five mixtures of spikes, each including 15 subsets of spikes.

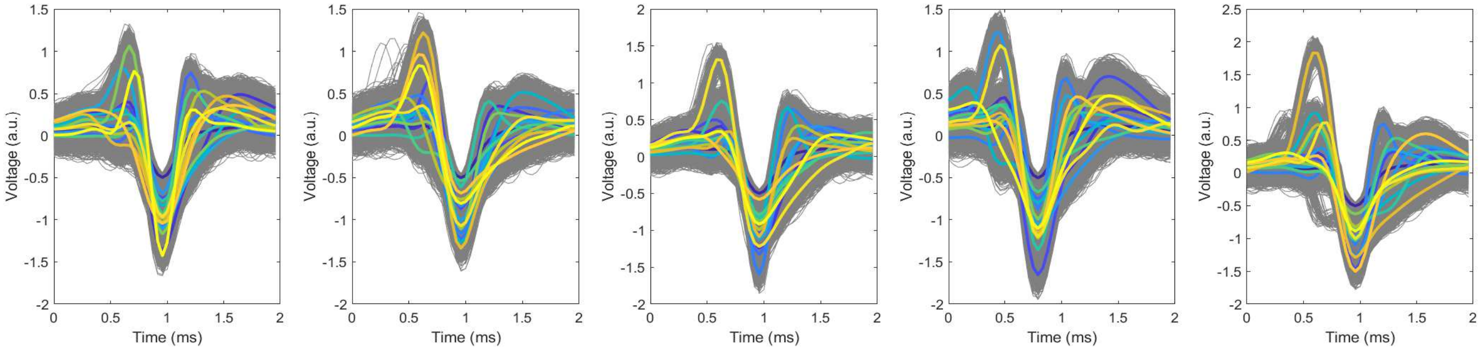


(n) Five mixtures of spikes, each including 16 subsets of spikes.

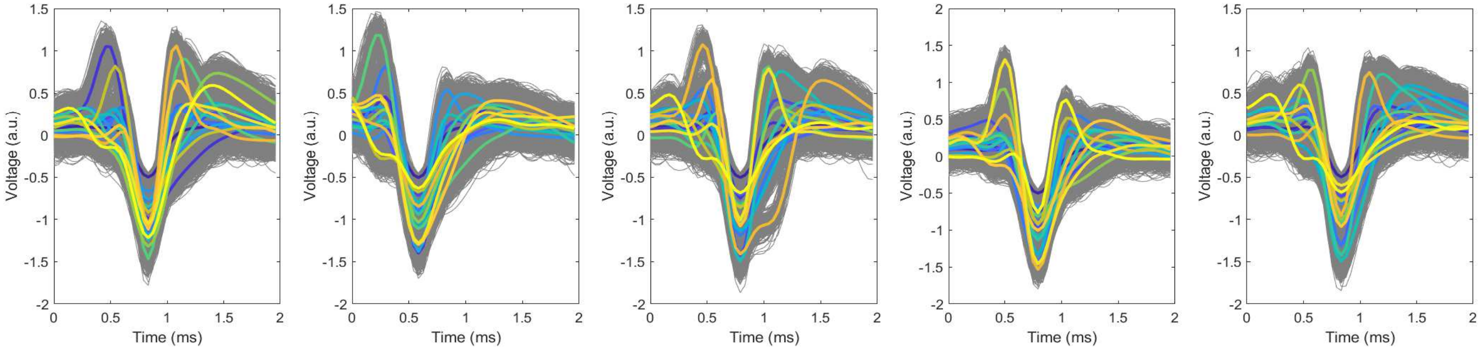


(o) Five mixtures of spikes, each including 17 subsets of spikes.

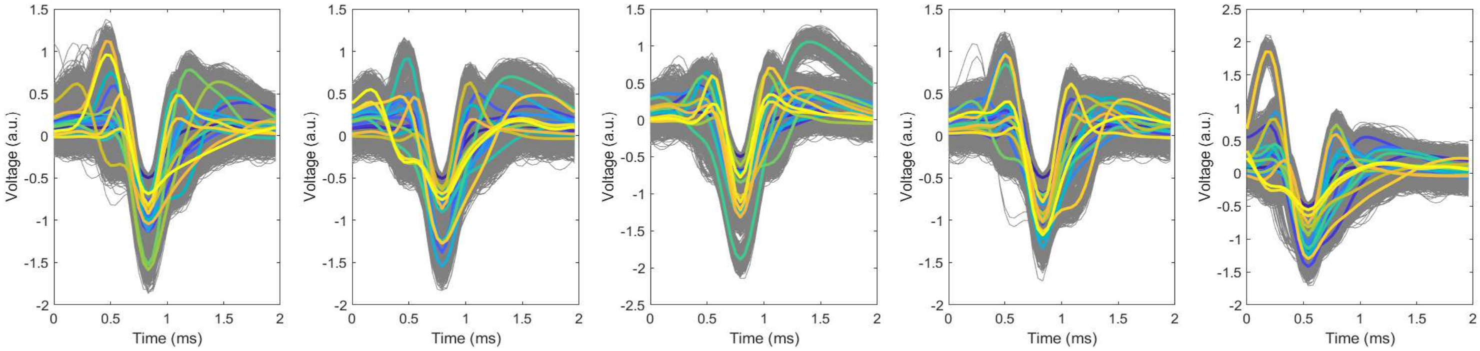


(p) Five mixtures of spikes, each including 18 subsets of spikes.

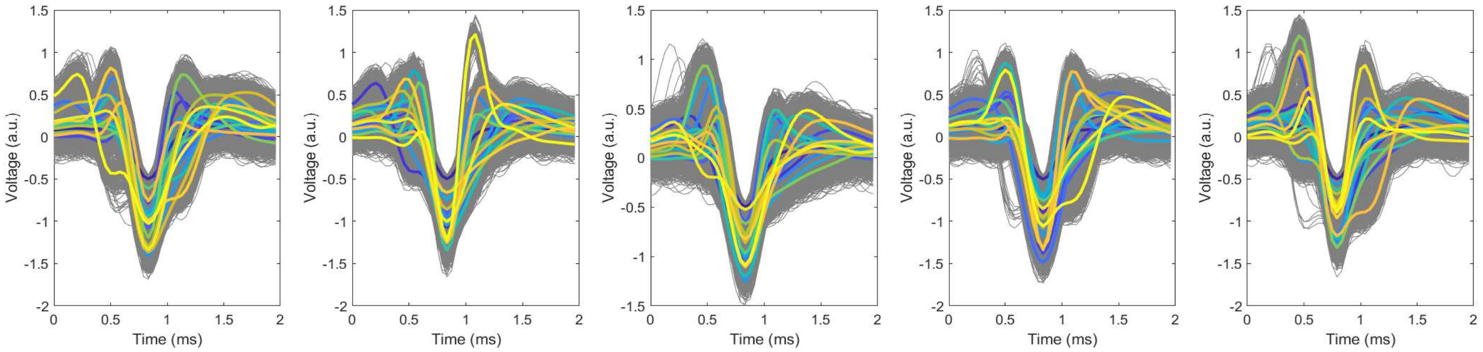


(q) Five mixtures of spikes, each including 19 subsets of spikes.

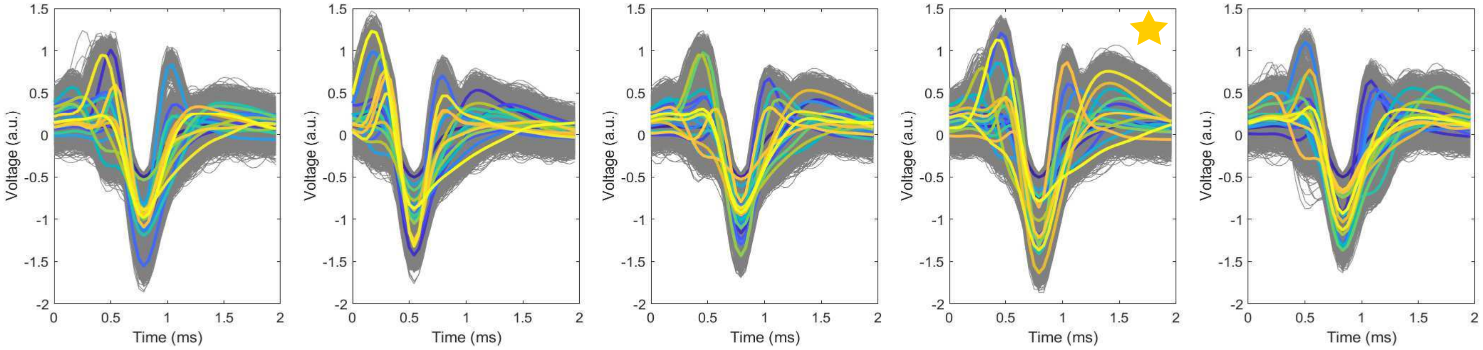


(r) Five mixtures of spikes, each including 20 subsets of spikes.
