# Supplementary material for: Cluster tendency assessment in neuronal spike data
Source: PLoS One. 2019 Nov 12;14(11):e0224547. doi: 10.1371/journal.pone.0224547 (PMC6850537; doi:10.1371/journal.pone.0224547)
Supplement: S3 Fig — In the PCA, Wavelet, t-SNE and Sammon plots, x axis is dimension 1 and y axis is dimension 2. In the PV-E plots, x axis is the peak to valley time (PV) in ms and y axis is the energy (E) of the waveform, unitless since derived from unitless simulations. (PDF) [file pone.0224547.s004.pdf]

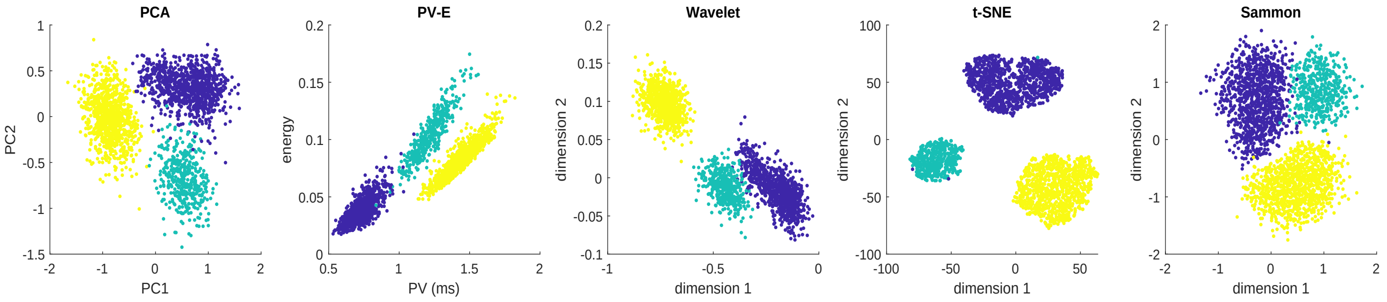

(a) Same projections as shown in Figure 1-a with axes labels

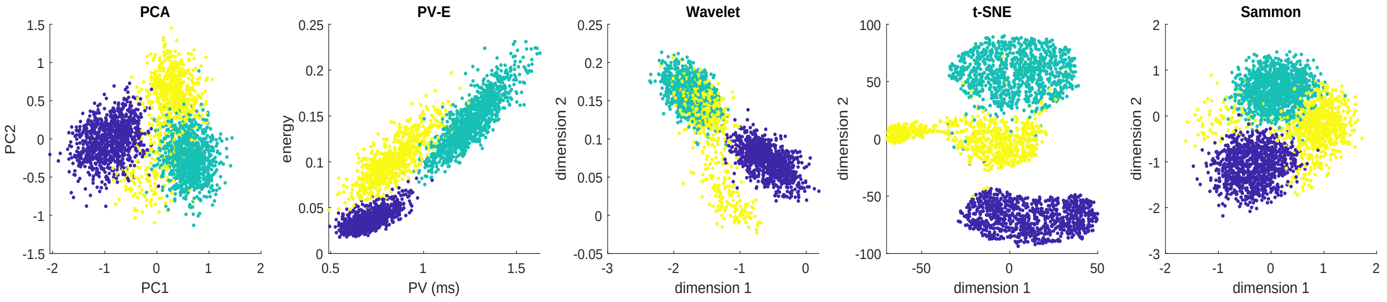

(b) Same projections as shown in Figure 1-b with axes labels

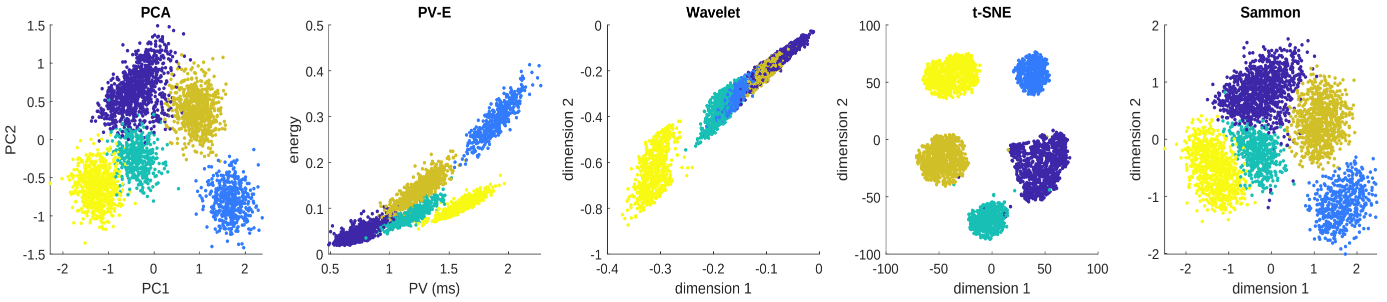

(c) Same projections as shown in Figure 2 with axes labels

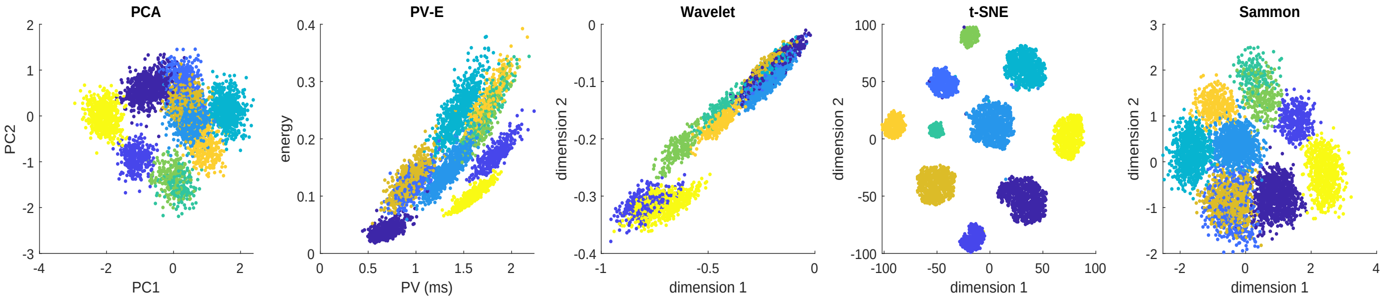

(d) Same projections as shown in Figure 3 with axes labels

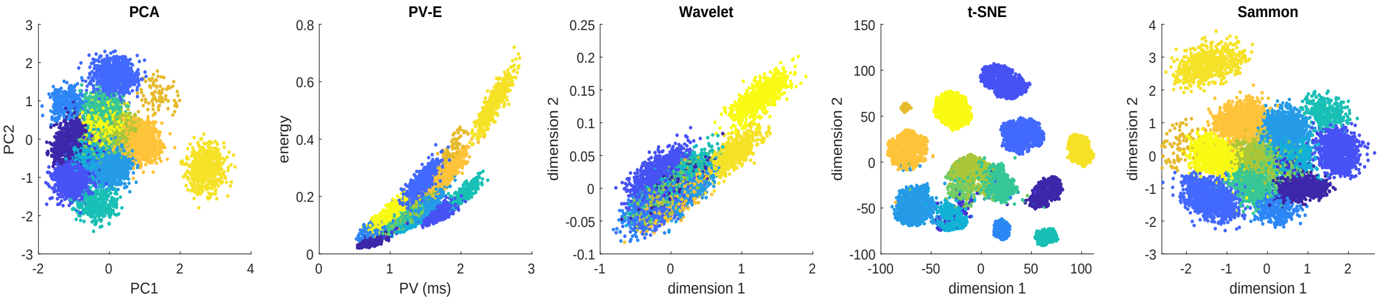

(e) Same projections as shown in Figure 4 with axes labels

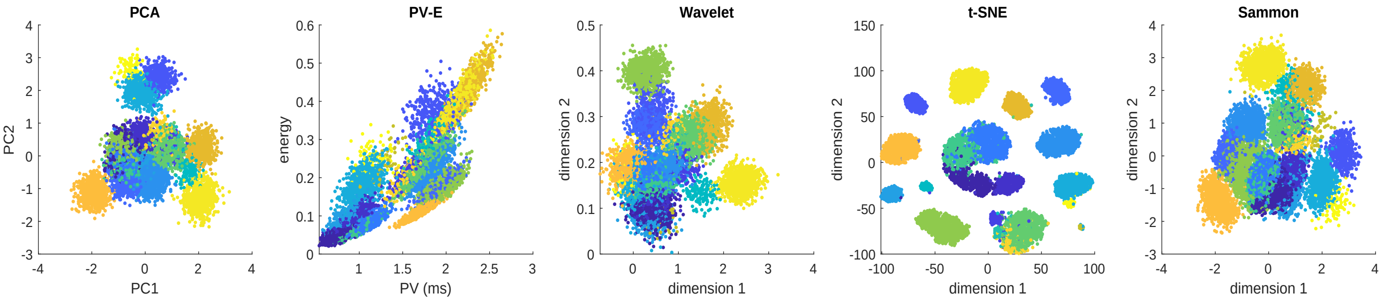

(f) Same projections as shown in Figure 5 with axes labels
