# Supplementary material for: Cluster tendency assessment in neuronal spike data
Source: PLoS One. 2019 Nov 12;14(11):e0224547. doi: 10.1371/journal.pone.0224547 (PMC6850537; doi:10.1371/journal.pone.0224547)
Supplement: S4 Fig — Those figures investigated all possible mixtures of the 4 subsets (i.e. sets of spikes fired by each neuron) shown in Fig 8. The subsets are taken from dataset-2 which are waveforms in microvolt units. In the PCA, Wavelet, t-SNE and Sammon plots, x axis is dimension 1 and y axis is dimension 2. In PV-E, x axis is peak to valley time (PV), in μs and y axis is energy (E) in μV2. (PDF) [file pone.0224547.s005.pdf]

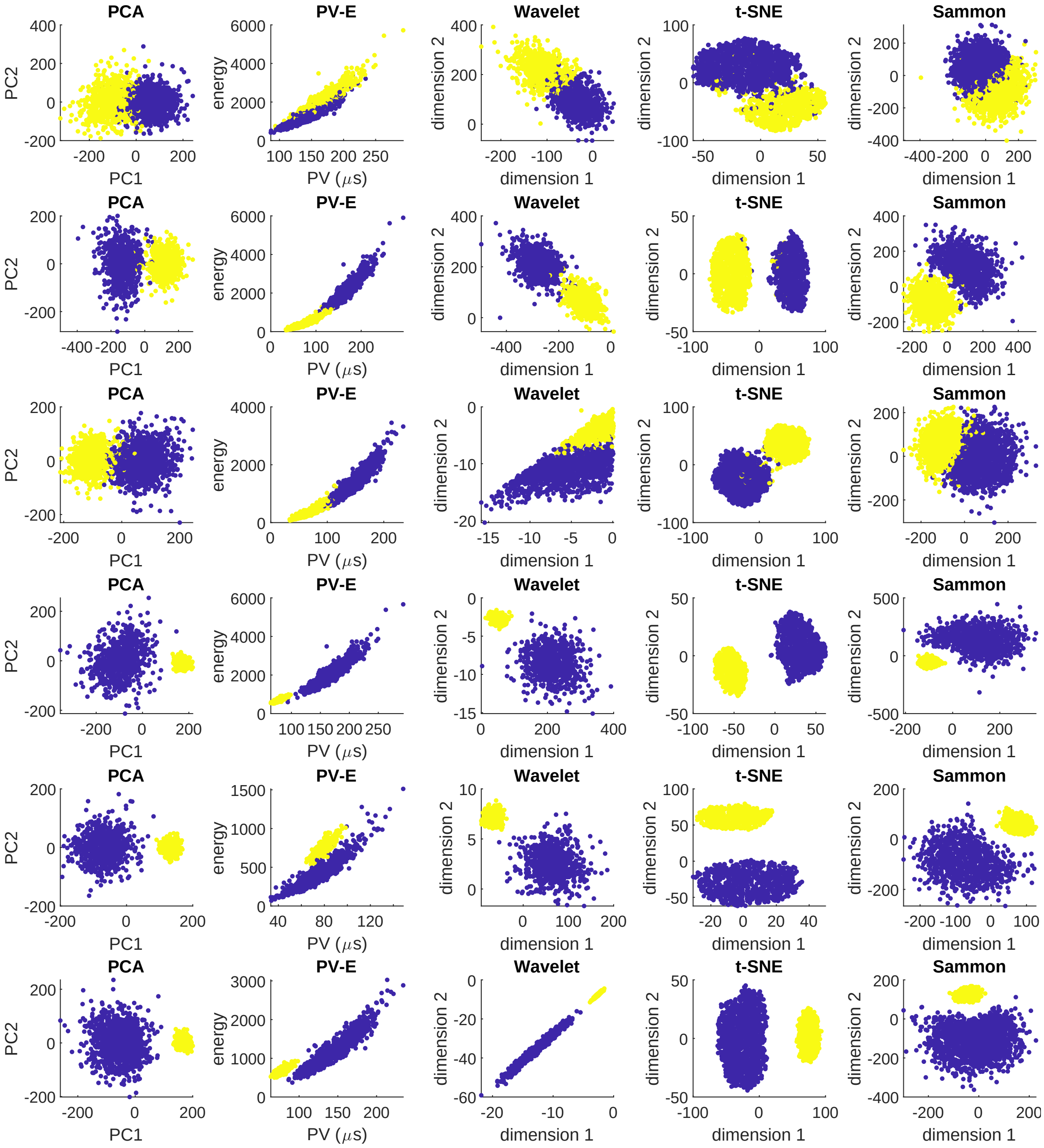

(a) Reproducing the projections in Figure 9 with labeled axis. Rows represent subfigures 9a to 9f

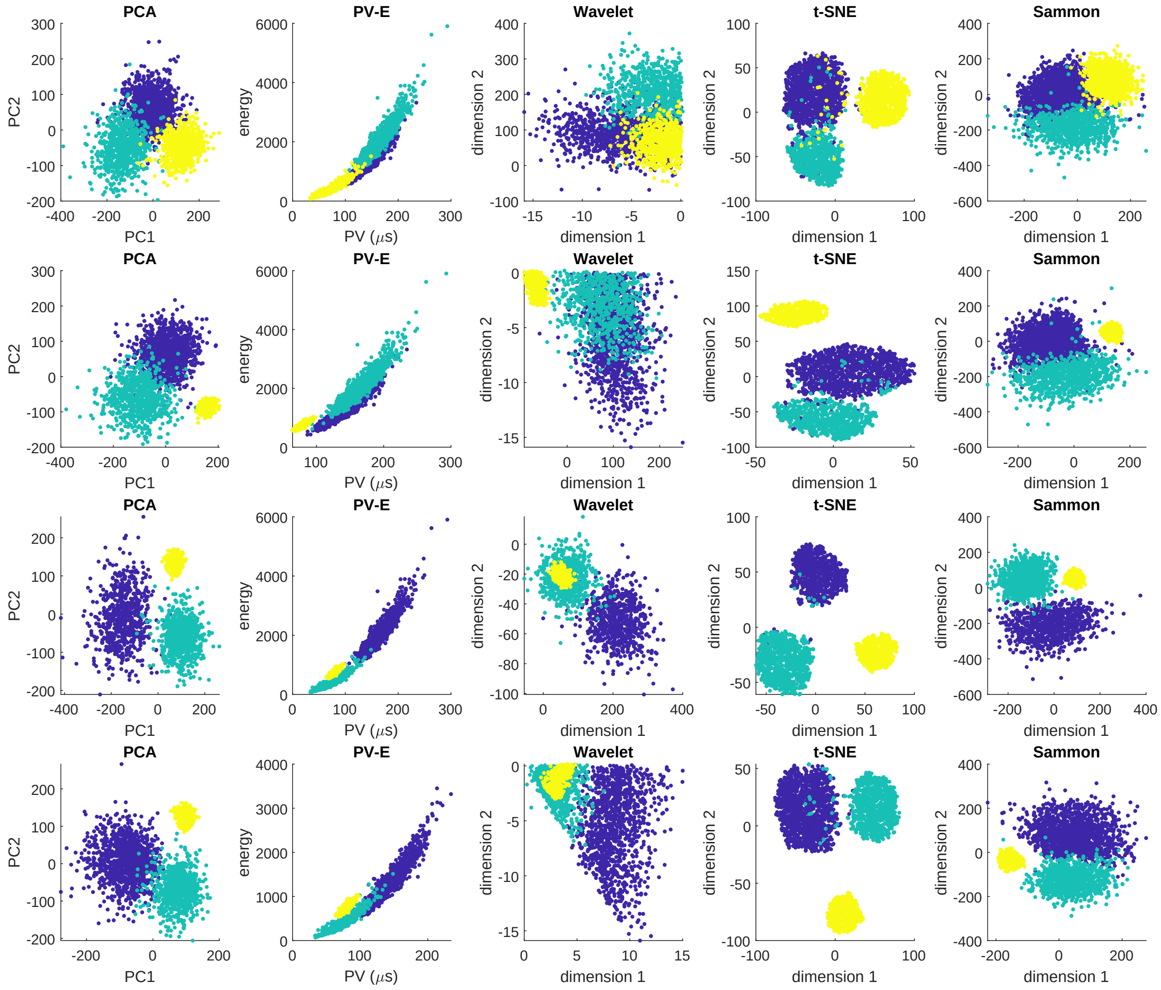

(b) Reproducing the projections in Figure 10 with labeled axis. Rows represent subfigures 10a to 10d

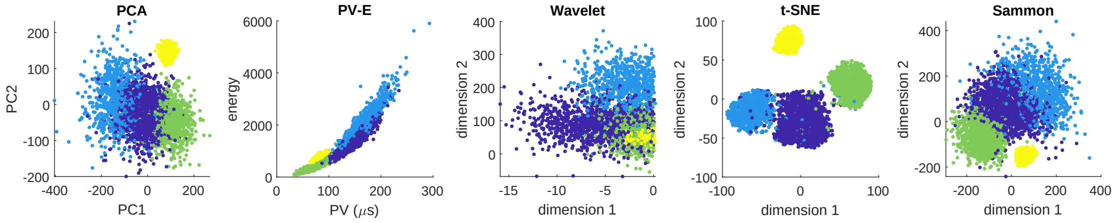

(c) Reproducing the projections in Figure 11 with labeled axis
